# Climatic Changes Lead to Declining Winter Chill for Fruit and Nut Trees in California during 1950–2099

**DOI:** 10.1371/journal.pone.0006166

**Published:** 2009-07-22

**Authors:** Eike Luedeling, Minghua Zhang, Evan H. Girvetz

**Affiliations:** 1 Department of Land, Air and Water Resources, University of California Davis, Davis, California, United States of America; 2 Department of Plant Sciences, University of California Davis, Davis, California, United States of America; 3 College of Forest Resources, University of Washington, Seattle, Washington, United States of America; University of Massachusetts Amherst, United States of America

## Abstract

**Background:**

Winter chill is one of the defining characteristics of a location's suitability for the production of many tree crops. We mapped and investigated observed historic and projected future changes in winter chill in California, quantified with two different chilling models (Chilling Hours, Dynamic Model).

**Methodology/Principal Findings:**

Based on hourly and daily temperature records, winter chill was modeled for two past temperature scenarios (1950 and 2000), and 18 future scenarios (average conditions during 2041–2060 and 2080–2099 under each of the B1, A1B and A2 IPCC greenhouse gas emissions scenarios, for the CSIRO-MK3, HadCM3 and MIROC climate models). For each scenario, 100 replications of the yearly temperature record were produced, using a stochastic weather generator. We then introduced and mapped a novel climatic statistic, “safe winter chill”, the 10% quantile of the resulting chilling distributions. This metric can be interpreted as the amount of chilling that growers can safely expect under each scenario. Winter chill declined substantially for all emissions scenarios, with the area of safe winter chill for many tree species or cultivars decreasing 50–75% by mid-21^st^ century, and 90–100% by late century.

**Conclusions/Significance:**

Both chilling models consistently projected climatic conditions by the middle to end of the 21^st^ century that will no longer support some of the main tree crops currently grown in California, with the Chilling Hours Model projecting greater changes than the Dynamic Model. The tree crop industry in California will likely need to develop agricultural adaptation measures (e.g. low-chill varieties and dormancy-breaking chemicals) to cope with these projected changes. For some crops, production might no longer be possible.

## Introduction

**Figure 1:**
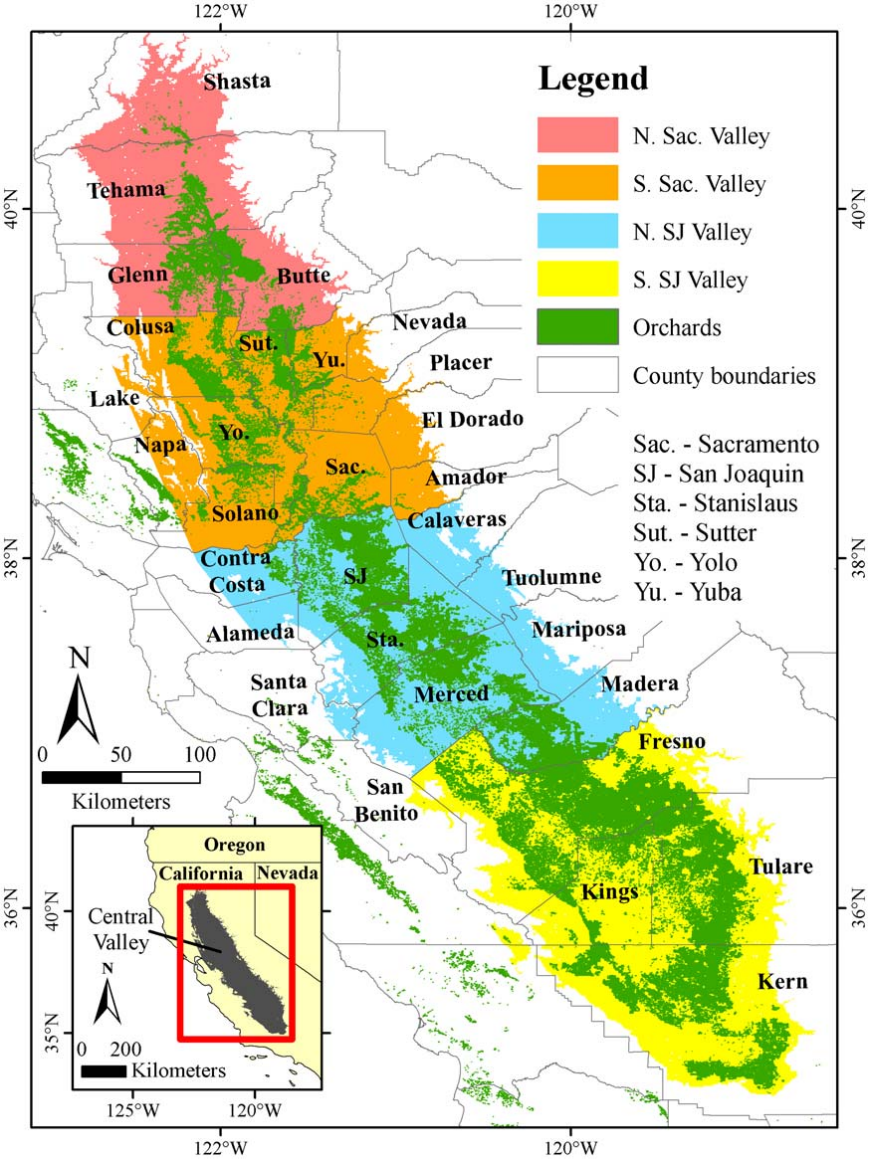
Overview of California's Central Valley, showing the distribution of orchards that require winter chill, the major producing counties and the subdivisions of the Central Valley analyzed separately in this study.

Cool temperatures in the winter are essential for successful cultivation of many tree crops [1]–[4]. All economically important fruit and nut tree species that originated from temperate and cool subtropical regions have chilling requirements that need to be fulfilled each winter to ensure homogeneous flowering and fruitset, and generate economically sufficient yields. The state of California (USA) alone is home to 1.2 million hectares of orchards cropped with trees that have chilling requirements (Fig. 1), supporting an estimated US$ 8.7 billion industry [5]. Due to its need for sufficient winter chill, this industry is vulnerable to the observed recent past and future projected increases in temperatures due to global climate change [6]–[8].

Insufficient winter chill can severely reduce crop yields and crop quality. When chilling requirements are not completely fulfilled, trees display irregular and temporally spread out flowering, leading to inhomogeneous crop development. This process ultimately results in varying crop sizes and maturity stages at the time of harvest, which can substantially reduce yield amount and value [4], [9]. For species that rely on overlap between male and female flowering, such as walnuts and pistachios, insufficient chilling can reduce pollination, also leading to reduced crop yields [10]. If winter chill decline occurs due to climate change, production constraints are likely to exceed those typically reported, because many trees might not even come close to fulfilling their chilling requirements. In such cases, complete crop failures may frequently occur, while early senescence of trees will further reduce their yield potential, rendering many orchard operations uneconomical [4], [11].

Agricultural scientists have developed mathematical models that are used by growers to select tree cultivars with chilling requirements that correspond to available chilling at a specific location. However, a grower's understanding of available winter chill is likely to reflect conditions of the past rather than those expected for a warmer future. Since orchards often remain in production for decades, consideration of future expected winter chill is necessary in times of imminent climatic changes. Without such considerations, many orchards might receive inadequate chilling by the time they reach physiological maturity, even though at the date of planting, climatic conditions were optimal for the chosen cultivars. Depending on the pace of winter chill decline, the consequences for California's fruit and nut industries could be devastating.

While a few studies have investigated the impact of climate change on winter chill [6]–[8], none have mapped the spatial extent of past and projected changes. Here we develop spatially continuous estimates of winter chill for the entire state of California. We use historic temperature data, as well as a range of climate change projections, to quantify winter chill with two different mathematical models, the Chilling Hours Model, which is predominantly used in California, and the Dynamic Model, which is often considered superior in subtropical climates [12]–[16]. While the Chilling Hours Model simply counts the number of hours during the winter, when temperatures are between 0 and 7.2°C [17], [18], the Dynamic Model assumes that winter chill is accumulated in a two-step process. According to the Dynamic Model, an intermediate chilling product is first formed by a process that requires cool temperatures. This intermediate product, whose formation is enhanced by moderate temperatures, can be destroyed by high temperatures. When a certain quantum of the intermediate product has accumulated, it is converted irreversibly into a Chill Portion [19], [20]. We introduce the concept of “safe winter chill”, the amount of chilling that can safely be expected in 90% of all years and quantify the change in area of safe winter chill for exemplary crop species. We expect this metric to be more useful for tree crop growers than mean winter chill, since it incorporates the economic need for an orchard operation to produce good yields in most years (90%), rather than in an average year.

For generating the hourly temperature records needed for quantifying winter chill from daily records, which are more readily available, we correlated short-term hourly with long-term daily temperature records by Partial Least Squares regression (for accuracy estimates refer to Supporting Information S1 and Figure S1). After stochastically generating synthetic 100-year daily temperature records representing climatic conditions in 1950, 2000, 2041–2060 and 2080–2099, we used the regression equations to estimate hourly temperatures. From this dataset, we estimated safe winter chill for each point in time, with future predictions based on three IPCC greenhouse gas emissions scenarios (B1, A1B and A2), and averaged over three global climate models (CSIRO-MK3, HadCM3 and MIROC). Point estimates of winter chill from all weather stations were then spatially interpolated.

## Results

### Mean vs. safe winter chill

**Figure 2:**
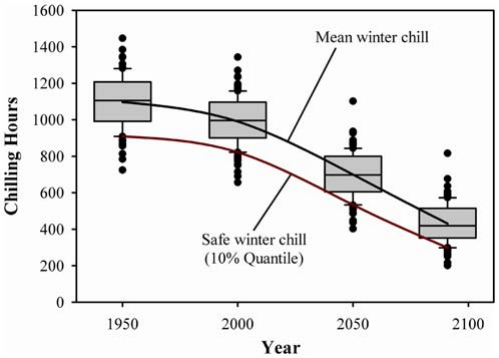
Distribution of annual winter chill estimates (in Chilling Hours) for Davis, CA, based on 100-year synthetic weather records for each point in time. In box plots, the central line indicates the median of the distribution, the edges of the boxes are the 25% and 75% quantiles, error bars are the 10% and 90% quantiles, and dots indicate outliers.

When using the Chilling Hours Model to calculate winter chill, safe winter chill was about 130 Chilling Hours lower than mean winter chill on average over all locations and scenarios analyzed (shown for Davis in Fig. 2). The gap between the two metrics decreased slightly over time, from 146 in 1950 to 131 in 2041–2060 and 115 in 2080–2099, tracing the general decline in winter chill. When using the Dynamic Model for chilling quantification, the difference between safe and mean winter chill remained approximately constant at 7.5 Chill Portions, with the early scenarios showing slightly smaller differences of 6.7 (1950) and 7.1 (2000) Chill Portions.

### Future winter chill in California

In all scenarios, winter chill in California declined substantially over time. The MIROC GCM projected the greatest warming and thus the greatest decreases in winter chill, followed by the HadCM3 and CSIRO GCMs. Since none of these models can be clearly identified as being more accurate than the others, we only show winter chill averaged over all three models. Supporting Figures S2 and S3 show safe winter chill for each point in time and IPCC emissions scenario analyzed, quantified with the Chilling Hours Model and the Dynamic Model, respectively. The Chilling Hours Model consistently projected greater losses than the Dynamic Model, confirming its greater sensitivity to rising temperatures [8].

**Figure 3:**
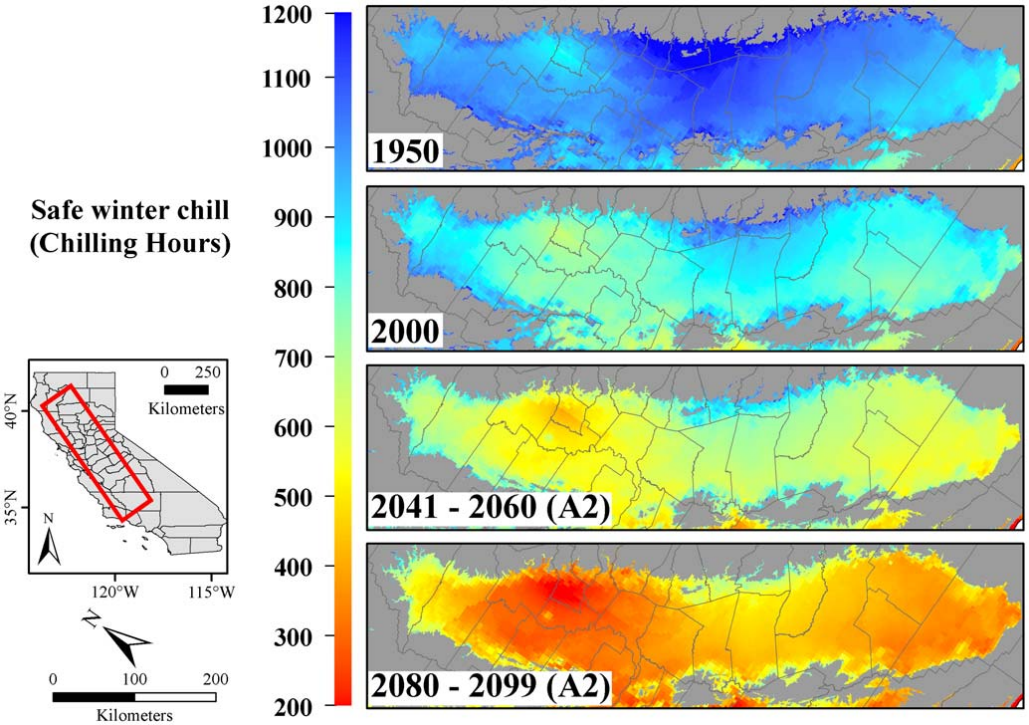
Safe winter chill in California's Central Valley in 1950, 2000, 2041–2060 and 2080–2099, calculated with the Chilling Hours Model. Future winter chill was quantified using the A2 IPCC greenhouse gas emissions scenario.

Since most of the state's fruit and nut production is located in the Central Valley (Fig. 1), winter chill projections are most crucial for this area. Our analysis showed that around the year 1950, growers in the Central Valley could rely on between 700 and 1200 Chilling Hours, depending on the location of their orchard in the valley (Fig. 3). By 2000, this number had already declined by up to 30% in some regions. Based on the A2 greenhouse gas emissions scenario (highest emissions scenario analyzed), winter chill is likely to decrease by 30–60% relative to 1950 by mid century, and by up to 80% by the end of the century.

**Figure 4:**
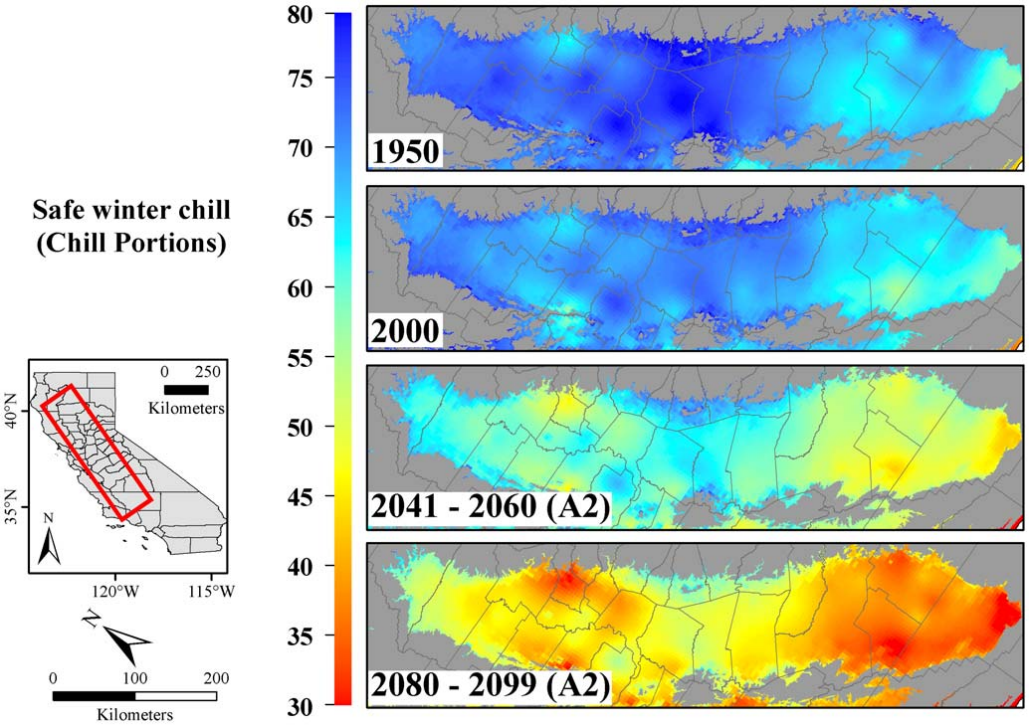
Safe winter chill in California's Central Valley in 1950, 2000, 2041–2060 and 2080–2099, calculated with the Dynamic Model. Future winter chill was quantified using the A2 IPCC greenhouse gas emissions scenario.

Changes projected by the Dynamic Model were less severe than for the Chilling Hours Model, but nevertheless likely to strongly impact fruit and nut production. By the end of the 21^st^ century, this model projected decreases in winter chill between 30 and 60% of 1950 conditions (Fig. 4).

Decreases in winter chill between 1950 and 2080–2099 varied geographically between the Northern Sacramento Valley (NSacV; Fig. 1), the Southern Sacramento Valley (SSacV; Fig. 1), the Northern San Joaquin Valley (NSJV; Fig. 1) and the Southern San Joaquin Valley (SSJV; Fig. 1) (Table 1). When using the Chilling Hours Model to quantify winter chill, chilling losses were most severe in the SSacV, which lost 51% (B1 scenario), 61% (A1B) and 67% (A2) compared to 1950 winter chill. Among the other regions (NSJV, SSJV and NSacV), winter chill decreases were similar at about 41% (B1), 50% (A1B) and 56% (A2). Use of the Dynamic Model led to different projections of 2080–2099 winter chill, showing maximum losses of 43% for the SSJV under the A2 emissions scenario, compared to 40% in the SSacV, 35% in the NSJV and 33% for the NSacV (Table 1).

**Table 1:** Means and standard deviations of safe winter chill modeled for four regions in California's Central Valley for 1950, 2000, 2041–2060* and 2080–2099*.

Year	1950	2000	Mid 21^st^ century (2041–2060)			End 21^st^ century (2080–2091)		
Emission scenario			B1	A1B	A2	B1	A1B	A2
	*Chilling Hours±Standard deviation (Chilling Hours Model)*							
Northern Sacramento Valley	993±43	870±70	697±91	647±100	654±100	577±112	498±125	439±137
Southern Sacramento Valley	1015±71	784±55	634±71	572±73	578±73	494±75	400±77	334±77
Northern San Joaquin Valley	1101±79	876±90	757±93	702±93	704±94	632±96	537±97	476±96
Southern San Joaquin Valley	979±70	844±58	697±69	647±70	649±71	587±71	489±74	423±74
	*Chill Portions±Standard deviation (Dynamic Model)*							
Northern Sacramento Valley	73.0±2.0	70.8±2.0	62.8±3.3	60.2±3.7	61.1±3.6	56.7±4.1	51.3±5.0	48.7±5.5
Southern Sacramento Valley	73.8±2.9	69.7±2.9	61.1±4.0	58.0±4.3	59.2±4.3	54.1±4.5	47.5±4.9	44.3±5.2
Northern San Joaquin Valley	74.8±3.3	71.4±2.4	63.6±3.4	60.5±3.6	62.0±3.6	57.4±3.7	51.4±4.0	48.7±4.1
Southern San Joaquin Valley	67.0±3.5	64.3±2.9	54.5±3.6	50.6±3.8	52.2±3.8	47.6±3.9	41.6±4.0	37.9±4.2

* Future projections were done for the B1 (low), A1B (moderate) and A2 (high) IPCC emission scenarios.

### Suitable areas for future fruit and nut production

Winter chill decline strongly affected the spatial extent of areas suitable for the cultivation of tree crops with chilling requirements. For cultivars requiring 200 Chilling Hours, such as low-chill almonds, winter chill conditions are unlikely to become critical by the end of the 21^st^ century (Fig. 3). For cultivars with chilling requirements of 500 Chilling Hours, only about 78% of the Central Valley was suitable for production by the end of the 21^st^ century under the B1 and A1B emissions scenarios, and only 39% under the A2 scenario. For cultivars requiring more than 700 hours of chilling, conditions deteriorated earlier, with only between 23 and 46% of the area remaining suitable for production by 2041–2060, and between 2 and 10% by 2080–2099, depending on the emissions scenario. Such a chilling requirement is commonly considered the minimum for the cultivation of walnuts, apricots, plums and most peaches and nectarines. For species with a higher chilling requirement of more than 1000 hours (e.g., apples, cherries and pears), only 4% of the area in the Central Valley was suitable in 2000, and virtually no areas remained suitable by 2041–2060 under any emissions scenario. In interpreting these range estimates, it should be noted that the currently used chilling requirements (in Chilling Hours) might not be valid in a warmer climate.

## Discussion

Observed historic and future projected temperature increases in California strongly decreased the availability of winter chill under all greenhouse gas emissions scenarios, regardless of the model used to quantify this important climatic parameter for fruit production. On a global scale, it is likely that most other growing regions of subtropical fruit and nut trees with chilling requirements will be similarly affected by declining winter chill. Our projections showed that for many tree crops that now cover large areas within the Central Valley, climatic conditions will become less suitable and in many cases potentially prohibitive for production. Areas where safe winter chill exists for growing walnuts, pistachios, peaches, apricots, plums and cherries (>700 Chilling Hours) are likely to almost completely disappear by the end of the 21^st^ century. For cultivars with chilling requirements above 1000 Chilling Hours, such as apples, cherries and pears, very few locations with safe chilling levels were found to exist today, and our modeling results project that virtually none will exist by mid century.

The resulting reductions in crop yield and quality could severely impact California's tree crop growers. According to the USDA Agricultural Census of 2002, the state had 38,693 fruit and nut orchard farms, covering 1.2 million hectares of land and driving a US$ 8.7 billion industry [5]. Predictive yield modeling based on accumulated winter chill is not advanced enough to precisely predict the economic losses of winter chill decline, but the effects will almost certainly be felt by growers of many crops. Especially for those growers specialized in producing high-chill species and cultivars, winter chill decline might make current production systems infeasible. We expect few tree crops to be unaffected by these changes, with almonds and pomegranates likely to experience the smallest deterioration in production conditions due to their low chilling requirements.

Given the long life spans of orchards compared to annual crops and the substantial investments required for orchard establishment, tree crop growers will be much more vulnerable to the long and medium term effects of climate change than growers of annual crops, making the development of predictive temperature models for tree crop yields crucial for strategic planning of orchard operations.

Improved orchard management might have potential for alleviating winter chill decline, since planting density, pruning practices and irrigation regime can influence orchard microclimate [21]–[23]. So far, we are not aware of growers using such measures for managing winter chill, but in the future, such management options might increase in importance. For many growers, transitioning to a different cultivar or species will also be an option. For most species, cultivars with a wide range of chilling requirements are available, providing some genetic potential for adaptation and further breeding to reduce chilling requirements. There is also a range of rest-breaking chemicals, which can be used to partially compensate for a lack of sufficient chilling in many crops, such as cherries [24]. This option has not been explored for most common species in California, however, and the success of such chemical application appears to be restricted to a narrow time window during the later stages of the dormancy period [23]. Early applications have been found ineffective, while late applications might damage buds and reduce yield potentials. Timing applications of dormancy-breaking agents thus requires extensive knowledge about the progression of trees through the dormancy period, and the availability of accurate winter chill models.

Research on chilling models in many subtropical regions has indicated that the Chilling Hours Model is not very precise in this climatic zone [12]–[16]. California cherry growers have therefore adopted the Dynamic Model, which works better for deciding when to apply dormancy-breaking agents. As temperatures rise, many more crop species are likely to benefit from using alternative chilling models, or from the development of crop-specific chilling models, based on experiments or analysis of historic phenological records [14]. Currently, the orchard industry is poorly prepared for such a transition, since chilling requirements are only available for the Chilling Hours Model, whose accuracy is likely to decrease with further temperature increases [8], [14]. For assisting growers in preparing for the consequences of climate change, and for averting detrimental effects on food security and farm economics, efforts to project climate impacts on tree crop production should be enhanced, including the development and testing of improved chilling models and management options to influence orchard microclimate.

While we are confident of the general trend of declining winter chill, some locations within the Central Valley might remain suitable even for crops with high chilling requirements. Locations with cooler microclimatic conditions might be found along major rivers, in the foothills of Sierra Nevada and Coastal Range, where cold air tends to drain, as well as close to the Sacramento Delta and in those parts of the Central Valley, where frequent fogs reduce temperatures during the winter. On the other hand, it is likely that warmer temperatures will reduce the incidence of fog in many places, leading locally to stronger deterioration in winter chill than projected in this study.

The high sensitivity of the commonly used Chilling Hours Model to climate change [8] complicates the estimation of future ranges for tree crops. It seems likely that chilling requirements established under modern or past climatic conditions will not remain valid in a warmer future [14]. At present, chilling requirements are only available in Chilling Hours for most species and cultivars, providing the only basis for projections of future ranges of tree crops (Fig. 3). More efforts need to be expended for establishing chilling requirements in units of the Dynamic Model, which is more likely to remain valid as the climate changes [8], [14]. Such updated requirements would greatly enhance the accuracy of future range estimates.

While this study focused only on winter chill, climate change may have other (positive and negative) effects on tree crop production. Rising summer temperatures can be expected to be beneficial to some crops, while having a negative impact on others [25], [26], accelerated spring warming might reduce fruit sizes [27], and the projected scarcity and increasing price of irrigation water might also affect the economics of tree production [28]. So far, efforts to predict how the various effects of climate change will play out for tree crop growers have not been undertaken, making projections about the future of such operations in California's Central Valley difficult.

## Materials and Methods

### Weather records

Hourly temperature records are required for estimating winter chill with all common methods without resorting to idealized daily temperature curves. We obtained records of hourly temperatures for all 205 (active and inactive) stations of the California Irrigation Management Information System [CIMIS; 29]. The length of the weather records in this database ranged from less than one year to almost 27 years, with 96 stations having more than 10 years of data. As input for the weather generator, we also obtained daily measurements of solar radiation from all available CIMIS stations.

Since the CIMIS network was only established in 1982, it is not very suitable for analyzing long-term climatic changes. We therefore obtained daily measurements of minimum and maximum temperatures and precipitation from all 113 weather stations in California that belong to the cooperative weather station network administered by the National Climatic Data Center [30]. For 97 of these stations, records were available since at least 1951, while five datasets only started in the 1960s and two in the 1970s.

### Pairing weather stations

For using daily measurements to estimate hourly temperatures, each CIMIS weather station was associated with a nearby NCDC station. Using the Euclidean Allocation function of a Geographical Information System (GIS; ArcGIS 9.2, ESRI, Redlands, CA, USA), each CIMIS station was assigned the closest weather station of the other network, resulting in pairs of weather stations that were on average 20 km apart (max. distance was 76 km). The daily and hourly datasets of these station pairs were then joined. To remove records that were considered faulty, all hourly temperature records that were more than 5°C above the daily maximum or below the daily minimum of the NCDC record were eliminated from the dataset.

### Estimating daily and hourly temperatures

When analyzing observed or modeled weather records, long-term trends are often obscured by interannual variation. Temperatures observed during a particular year are often substantially warmer or cooler than the long-term running average for that year. This constraint can be overcome by generating synthetic weather records, which allow correction for interannual variation and facilitate statistical evaluation of weather records [7]. In creating synthetic weather records, the site-specific variation of important climatic parameters is evaluated and expressed in statistical terms, e.g. as the standard deviation of daily temperatures from the monthly mean, or the average duration of wet and dry spells [31]. Based on these statistical characteristics, specialized software can generate replicates of a given year. Variation in such a record is introduced by a random seed.

We used the LARS-WG stochastic weather generator [31] to produce synthetic 100-year weather records for each of our climate scenarios. This software computes site-specific weather statistics based on daily minimum and maximum temperatures, precipitation and solar radiation. It then uses these estimates to generate daily records of these parameters, based on user-defined climate change scenarios.

Since all common winter chill models require hourly temperatures as inputs, such records had to be derived from the daily datasets. To establish a relationship between daily and hourly temperatures, we performed separate Partial Least Squares [PLS; 32], [33] regression analyses for each station pair and each hour of the day [7], [8]. Independent variables in these regressions were the daily minimum temperature (T_min_), daily maximum temperature (T_max_) and daylength (DL), where T_min_ and T_max_ were measured at the weather stations, and DL was modeled for each day of the record [34], [35]. The PLS regression equations were used to explain the variation in hourly temperatures observed during the day.

In order to achieve the most accurate predictive equations, we used a cross-validation procedure (JMP 7, SAS Institute, Cary, NC, USA) to identify the most appropriate dimension for the regression models. This procedure splits the dataset into two or more groups and fits a regression model to all groups except one. The resulting model is then used to predict the values in the omitted group. This process is repeated for all groups and errors are quantified, providing an estimate of overall model accuracy. The number of latent factors is then chosen to maximize the overall accuracy in estimating hourly temperatures.

### Projecting temperatures under climate scenarios

Two climate scenarios representing 1950 and 2000 conditions were based on temperatures observed during the historic record. To obtain representative conditions for these two years, we calculated separate linear regression analyses for each month of the year from the entire daily temperature record for each NCDC weather station that was used to estimate hourly temperatures. Regressions were calculated for minimum and maximum daily temperatures, as well as for daily precipitation. Based on the resulting equations, representative values for all three parameters were obtained for both 1950 and 2000, and converted into separate climate scenario input files for LARS-WG for each weather station pair.

Future winter chill conditions were estimated based on statistically downscaled climate projections for minimum and maximum daily temperatures (averaged monthly) from three General Circulation Models—UKMO-HadCM3, CSIRO-MK3.0, and MIROC3.2(medres)—each run under the SRES A2, A1B, and B1 greenhouse gas emissions scenarios from the Intergovernmental Panel on Climate Change AR4 [36]. These nine future projections (three models by three emissions scenarios, developed by R. Neilson and MAPSS group, unpublished data) had been statistically downscaled to a 5 arc-minute resolution using the PRISM climate dataset (http://www.prism.oregonstate.edu) to calibrate the downscaling. Then the average minimum daily and maximum daily temperatures for each month during 2041–2060 and during 2080–2099 were calculated for each of the CIMIS weather station locations using the ClimateWizard climate-change analysis toolbox (http://ClimateWizard.org; Girvetz et al., in preparation). A twenty-year period was averaged to give a robust estimate of temperatures around 2050 (mid 21^st^ century) and around 2090 (late 21^st^ century) that is not influenced by year-to-year fluctuations in the projected climate.

### Winter chill models

We calculated winter chill according to two methods that are currently used in California. The most common chilling model used in the state is the Chilling Hours Model [sometimes referred to as Weinberger Model; 17,18]. In this model, chilling is quantified by simply adding up all hours, during which temperatures range between 0 and 7.2°C [refer to ref. 8 for equations describing both models]. As commonly practiced in California, we quantified accumulated winter chill between Nov 1^st^ and Mar 1^st^ of each winter season.

In recent years, growers of cherries in California have adopted the Dynamic Model, developed in Israel [19], [20], [37], to measure winter chill. In this model, chilling is assumed to accumulate as the result of a two-step process. First, an intermediate product is accumulated in a process which requires cool temperatures and is promoted by intermittent moderate temperatures. As soon as a certain quantum of the intermediate has been accumulated, it is irreversibly converted into a Chill Portion. Warm temperatures can destroy the intermediate, but do not affect accumulated Chill Portions. This model has been adopted by the meteorological services of Israel, South Africa and some states in the southern United States [15]. While testing of the Dynamic Model has been limited in California, research in Israel [37], [38], South Africa [13], Spain [16] and Chile [15] has shown the Dynamic Model to be the best available winter chill model for warm subtropical climates. In contrast to the Chilling Hours Model, the Dynamic Model does not require setting arbitrary end or start dates for the winter season, because the reversibility of the accumulation process for the intermediate chilling product prevents accumulation of Chill Portions when daily maximum temperatures are too high.

### Chilling metrics

For each time period analyzed (1950, 2000, 2041–2060, and 2080–2099), we calculated winter chill for 100 replications of each year, which allowed statistical evaluation of winter chill estimates. That is, rather than simply producing one value representing the winter chill accumulated during a given year, we used the variability produced by the stochastic weather generator to evaluate the distribution of winter chill over 100 replications of that year. This provided the ability to estimate the percentage of years, during which particular amounts of winter chill are likely to be available to fruit and nut growers.

While trends in winter chill are often analyzed using the mean of the chilling distribution, this measure is of subordinate importance to growers, because they economically depend on obtaining good yields in most (e.g. 90%) years rather than in an average year. Inadequate winter chill as often as once in ten years can threaten the economic sustainability of a farming operation. Here we present a novel climate change metric called “safe winter chill”, which we define as the 10% quantile of the winter chill distribution. This metric specifies the maximum chilling requirement that will be fulfilled in 90% of all years at a given site. In addition to calculating the mean of the winter chill distribution, we also calculated this safe winter chill metric.

### Spatial interpolation

Using the procedure outlined above, we estimated safe winter chill for all twenty climate scenarios at all suitable CIMIS weather stations in California. This procedure provided point estimates of safe winter chill, which needed to be interpolated to cover all of the state. We used the Kriging interpolation technique [with a spherical semivariogram, variable search radius and based on the 12 nearest data points; ref. 39] to create winter chill surfaces at a 20 arc-minute spatial resolution. While the resulting surface should fairly accurately describe safe winter chill in the relatively flat Central Valley, its validity in more mountainous terrain is limited, because elevation has a strong effect on winter chill and many locations are at substantially lower or higher elevations than the closest CIMIS station. To adjust for this effect, we estimated the elevation error of the interpolated surface by generating a Kriging surface through all point elevations of the weather stations. This surface was then subtracted from a Digital Elevation Model of California [SRTM30; ref. 40].

We then estimated the rate at which safe winter chill increases with increasing elevation separately for each climate scenario by calculating simple linear regressions between estimated safe winter chill and elevation across all weather stations. While the resulting regression equations were relatively poorly defined (coefficients of determination of <0.1 in many cases), all regressions were statistically significant at p<0.05, and their slopes should represent a reasonable estimate of the additional effect of elevation on safe winter chill. Multiplying the resulting rates with the elevation error surface and adding the results onto the original interpolated chilling grids produced error-adjusted safe winter chill surfaces for the entire state for each climate scenario.

For easier interpretation of the results, we finally created average surfaces for the chilling estimates resulting from the different General Circulation Models. This process resulted in eight winter chill surfaces, representing climatic conditions in 1950, 2000 and in 2041–2060 and 2080–2099 under the B1, A1B and A2 greenhouse gas emissions scenarios, respectively. To facilitate data processing, we implemented most analysis steps in JSL, the scripting language of JMP 7 and in the ArcGIS ModelBuilder.

## Supporting Information

Supporting Information S1 Accuracy estimate of winter chill projections. This text file describes the methods used to assess the accuracy of interpolated winter chill surfaces. Results are displayed in Figure S2.
(0.05 MB PDF)

Figure S1 Error estimates of projected winter chill. Qualitative error estimates of winter chill projections caused by elevation differences between the interpolated location and the closest CIMIS station (a) and by distance to the closest station (b).
(1.54 MB TIF)

Figure S2 Safe winter chill throughout California (in Chilling Hours). Safe winter chill (10% quantile of distribution over 100 modeled repetitions for each year) in California, quantified with the Chilling Hours Model for eight climate scenarios, representing climate conditions observed around 1950 and 2000, and projected for 2041–2060 and 2080–2099 under the B1, A1B and A2 IPCC greenhouse gas emissions scenarios.
(4.70 MB TIF)

Figure S3 Safe winter chill throughout California (in Chill Portions). Safe winter chill (10% quantile of distribution over 100 modeled repetitions for each year) in California, quantified with the Dynamic Model for eight climate scenarios, representing climate conditions observed around 1950 and 2000, and projected for 2041–2060 and 2080–2099 under the B1, A1B and A2 IPCC greenhouse gas emissions scenarios.
(4.63 MB TIF)

## References

[1] Samish RM (1954). Dormancy in Woody Plants.. Annual Review of Plant Physiology and Plant Molecular Biology.

[2] Knight TA (1801). Account of Some Experiments on the Ascent of the Sap in Trees.. Philosophical Transactions of the Royal Society of London.

[3] Saure MC (1985). Dormancy Release in Deciduous Fruit Trees.. Horticultural Reviews.

[4] Vegis A (1961). Samenkeimung und vegetative Entwicklung der Knospen.. Handbuch der Pflanzenphysiologie - Encyclopedia of Plant Physiology.

[5] USDA (2004). 2002 Census of Agriculture - United States - Summary and State Data..

[6] Baldocchi D, Wong S (2008). Accumulated winter chill is decreasing in the fruit growing regions of California.. Climatic Change.

[7] Luedeling E, Gebauer J, Buerkert A (in press). Climate change effects on winter chill for tree crops with chilling requirements on the Arabian Peninsula.. Climatic Change.

[8] Luedeling E, Zhang M, Luedeling V, Girvetz EH (in press). Sensitivity of winter chill models for fruit and nut trees to climate change.. Agriculture, Ecosystems and Environment.

[9] Chandler WH (1942). Deciduous orchards..

[10] Gradziel TM, Lampinen B, Connell JH, Viveros M (2007). ‘Winters’ almond: An early-blooming, productive, and high-quality pollenizer for ‘Nonpareil’.. Hortscience.

[11] Oukabli A, Mahhou A (2007). Dormancy in sweet cherry (Prunus avium L.) under Mediterranean climatic conditions.. Biotechnologie Agronomie Societe et Environnement.

[12] Erez A, Fishman S (1998). The dynamic model for chilling evaluation in peach buds.. Acta Horticulturae (ISHS).

[13] Linsley-Noakes GC, Allan P (1994). Comparison of 2 Models for the Prediction of Rest Completion in Peaches.. Scientia Horticulturae.

[14] Luedeling E, Zhang M, McGranahan G, Leslie C (submitted). Comparison of winter chill models for explaining walnut phenology.. Agricultural and Forest Meteorology.

[15] Perez FJ, Ormeno NJ, Reynaert B, Rubio S (2008). Use of the dynamic model for the assessment of winter chilling in a temperate and a subtropical climatic zone of Chile.. Chilean Journal of Agricultural Research.

[16] Ruiz D, Campoy JA, Egea J (2007). Chilling and heat requirements of apricot cultivars for flowering.. Environmental and Experimental Botany.

[17] Bennett JP (1949). Temperature and bud rest period.. California Agriculture.

[18] Weinberger JH (1950). Chilling Requirements of Peach Varieties.. Proceedings of the American Society for Horticultural Science.

[19] Fishman S, Erez A, Couvillon GA (1987). The Temperature-Dependence of Dormancy Breaking in Plants - Computer-Simulation of Processes Studied under Controlled Temperatures.. Journal of Theoretical Biology.

[20] Fishman S, Erez A, Couvillon GA (1987). The Temperature Dependence of Dormancy Breaking in Plants: Mathematical Analysis of a Two-Step Model Involving a Cooperative Transition.. Journal of Theoretical Biology.

[21] Sharma RR, Singh R (2006). Pruning intensity modifies canopy microclimate, and influences sex ratio, malformation incidence and development of fruited panicles in ‘Amrapali’ mango (Mangifera indica L.).. Scientia Horticulturae.

[22] Anconelli S, Facini O, Marletto V, Pitacco A, Rossi F Micrometeorological test of microsprinklers for frost protection of fruit orchards in Northern Italy; 2002..

[23] Erez A (1995). Means to compensate for insufficient chilling to improve bloom and leafing.. Acta Horticulturae.

[24] de Salvador FR, di Tommaso G (2003). Dormancy control in cherry.. Informatore Agrario.

[25] Lobell DB, Field CB, Cahill KN, Bonfils C (2006). Impacts of future climate change on California perennial crop yields: Model projections with climate and crop uncertainties.. Agricultural and Forest Meteorology.

[26] Lobell DB, Cahill KN, Field CB (2007). Historical effects of temperature and precipitation on California crop yields.. Climatic Change.

[27] Lopez G, Johnson RS, DeJong TM (2007). High spring temperatures decrease peach fruit size.. California Agriculture.

[28] Purkey DR, Joyce B, Vicuna S, Hanemann MW, Dale LL (2008). Robust analysis of future climate change impacts on water for agriculture and other sectors: a case study in the Sacramento Valley.. Climatic Change.

[29] CIMIS (2008). California Irrigation Management Information System..

[30] NCDC (2008). Database of the National Climatic Data Center. National Oceanic and Atmospheric Administration.

[31] Semenov MA (2008). Simulation of extreme weather events by a stochastic weather generator.. Climate Research.

[32] Wold S, van der Waterbeemd H (1995). PLS for multivariate linear modeling.. Chemometric methods in molecular design: methods and principles in medicinal chemistry.

[33] Wold S, Sjostrom M, Eriksson L (2001). PLS-regression: a basic tool of chemometrics.. Chemometrics and Intelligent Laboratory Systems.

[34] Spencer JW (1971). Fourier series representation of the position of the Sun.. Search.

[35] Almorox J, Hontoria C, Benito M (2005). Statistical validation of daylength definitions for estimation of global solar radiation in Toledo, Spain.. Energy Conversion and Management.

[36] IPCC (2007). Climate Change 2007 - Synthesis Report. Contributions of Working Groups I, II and III to the Fourth Assessment Report of the Intergovernmental Panel on Climate Change..

[37] Erez A, Fishman S, Linsley-Noakes GC, Allan P (1990). The dynamic model for rest completion in peach buds.. Acta Horticulturae (ISHS).

[38] Erez A, Fishman S, Gat Z, Couvillon GA (1988). Evaluation of winter climate for breaking bud rest using the dynamic model.. Acta Horticulturae (ISHS).

[39] Legendre P, Fortin MJ (1989). Spatial pattern and ecological analysis.. Vegetatio.

[40] GLCF (2008). Earth Science Data Interface at the Global Land Cover Facility..

