# Supplementary material for: Climatic Changes Lead to Declining Winter Chill for Fruit and Nut Trees in California during 1950–2099
Source: PLoS One. 2009 Jul 22;4(7):e6166. doi: 10.1371/journal.pone.0006166 (PMC2707005; doi:10.1371/journal.pone.0006166)
Supplement: Supporting Information S1 — Accuracy estimate of winter chill projections. This text file describes the methods used to assess the accuracy of interpolated winter chill surfaces. Results are displayed in Figure S2. (0.05 MB PDF) [file pone.0006166.s001.pdf]

Supporting Information S1: Accuracy estimate of winter chill projections

Partial Least Squares regression proved very successful in predicting hourly temperatures at all locations from hour of day, daylength and daily minimum and maximum temperatures at the closest NCDC weather station. On average over all locations, the coefficient of determination (r^2) of the resulting regression equations was 0.967, indicating that these four factors explained most of the temperature variation. This accuracy was deemed sufficient for projecting winter chill, given the uncertainty inherent in all projections of future climate change effects.

The accuracy of winter chill estimates is likely to vary throughout the state. The elevation difference between actual topography and the elevation surface extending through the point elevations of the CIMIS stations ranged from 0 to 2000 m (Fig. S2a), while the distance to the closest CIMIS station exceeded 200 km in some parts of California (Fig. S2b). Throughout the Central Valley, where most of the orchard area is located, both of these qualitative error estimates were small, indicating that winter chill projections should be fairly accurate for that area.
