# Supplementary figures and images for: Climatic Changes Lead to Declining Winter Chill for Fruit and Nut Trees in California during 1950–2099
Source: PLoS One. 2009 Jul 22;4(7):e6166. doi: 10.1371/journal.pone.0006166 (PMC2707005; doi:10.1371/journal.pone.0006166)

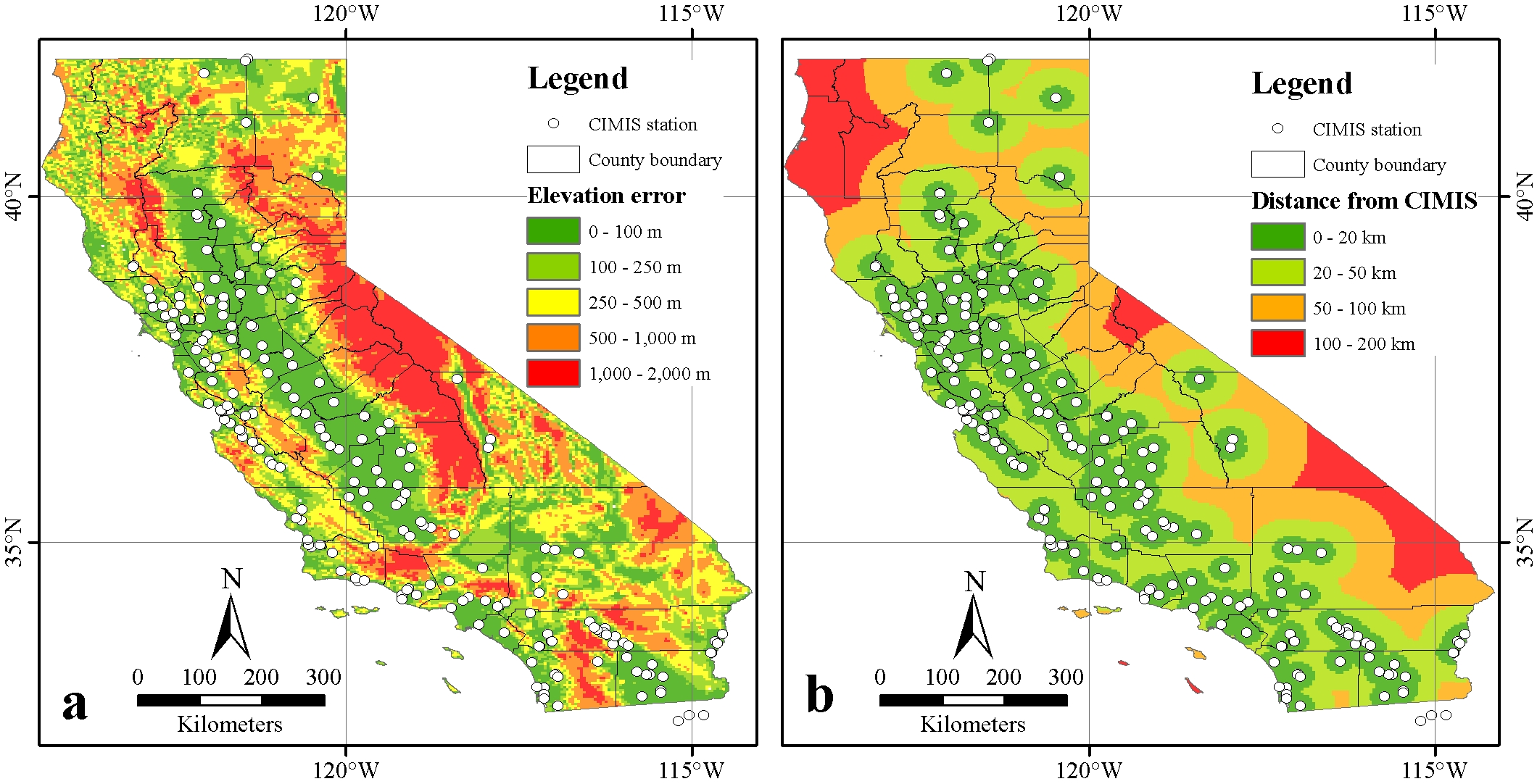

Supplement: Figure S1 — Error estimates of projected winter chill. Qualitative error estimates of winter chill projections caused by elevation differences between the interpolated location and the closest CIMIS station (a) and by distance to the closest station (b). (1.54 MB TIF) [file pone.0006166.s002.tif]

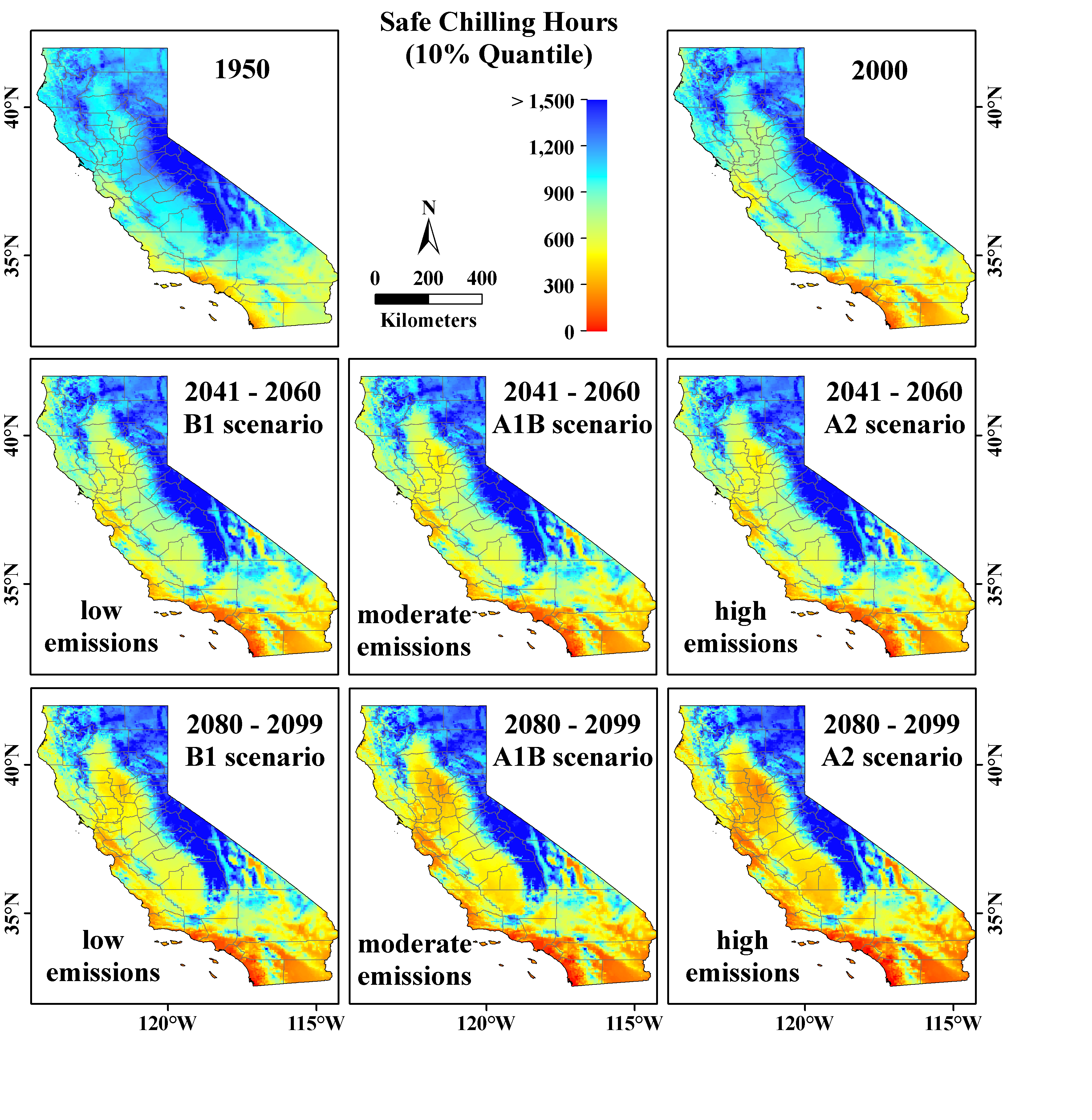

Supplement: Figure S2 — Safe winter chill throughout California (in Chilling Hours). Safe winter chill (10% quantile of distribution over 100 modeled repetitions for each year) in California, quantified with the Chilling Hours Model for eight climate scenarios, representing climate conditions observed around 1950 and 2000, and projected for 2041–2060 and 2080–2099 under the B1, A1B and A2 IPCC greenhouse gas emissions scenarios. (4.70 MB TIF) [file pone.0006166.s003.tif]

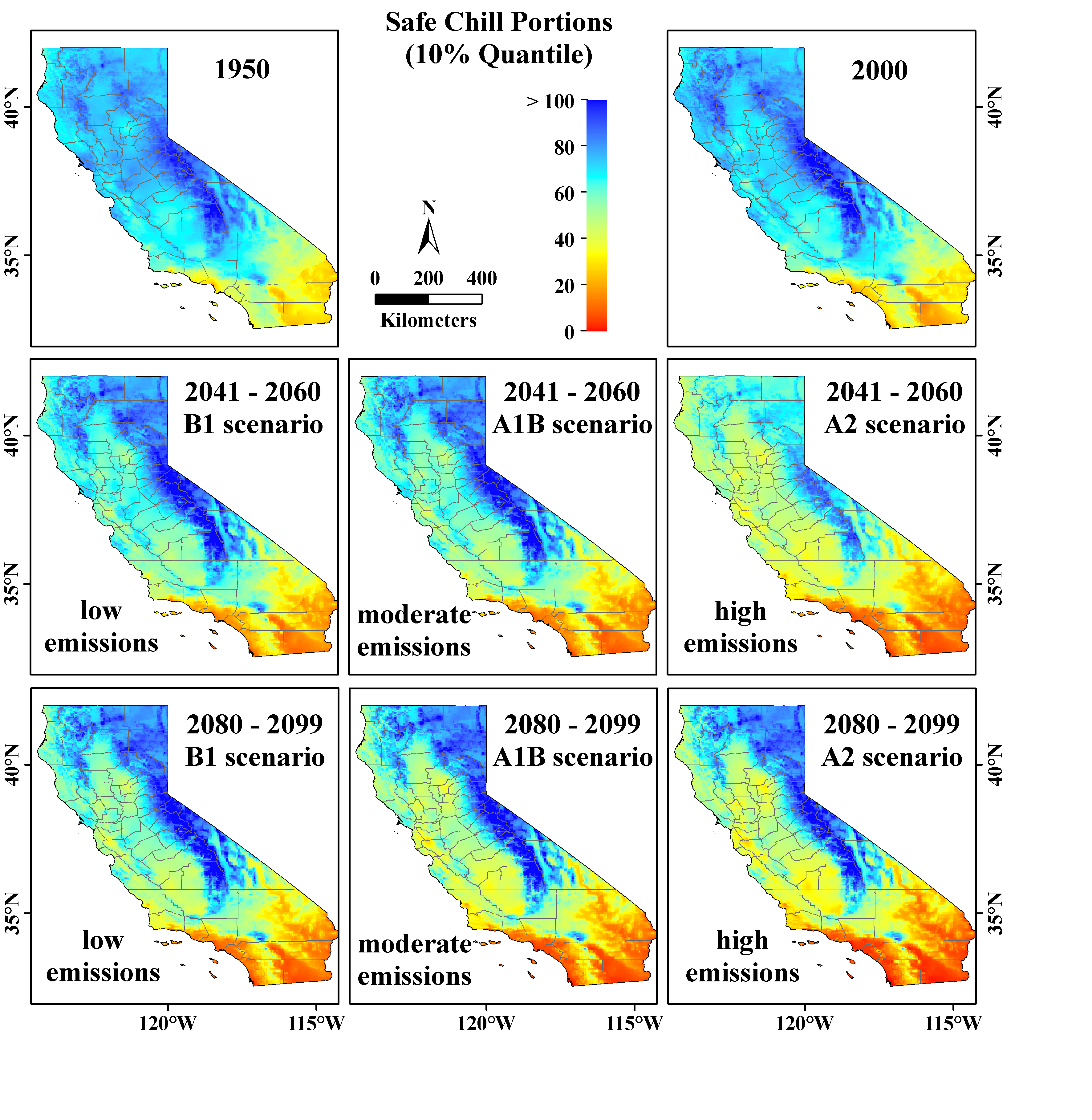

Supplement: Figure S3 — Safe winter chill throughout California (in Chill Portions). Safe winter chill (10% quantile of distribution over 100 modeled repetitions for each year) in California, quantified with the Dynamic Model for eight climate scenarios, representing climate conditions observed around 1950 and 2000, and projected for 2041–2060 and 2080–2099 under the B1, A1B and A2 IPCC greenhouse gas emissions scenarios. (4.63 MB TIF) [file pone.0006166.s004.tif]
